# Becoming a leader for underserved patients—the importance of student run free clinics

**DOI:** 10.3389/frhs.2024.1509964

**Published:** 2024-12-18

**Authors:** Ann Marie Cheney, Noah Baltrushes, Daniel Gehlbach, Armando Navarro

**Affiliations:** ^1^ Department of Social Medicine Population and Public Health, University of California Riverside, School of Medicine, Riverside, CA, United States; ^2^ Undergraduate Medical Education, University of California Riverside, School of Medicine, Riverside, CA, United States; ^3^ Family Medicine, University of California Los Angeles, School of Medicine, Los Angeles, CA, United States; ^4^ Family & Community Medicine, University of California San Francisco, School of Medicine, San Francisco Angeles, CA, United States

**Keywords:** student run free clincis, underserved patients, leadership, structural inequities, culturally competent care

## Abstract

Many students enter medical school with aspirations of expanding healthcare to underserved communities and reducing healthcare access barriers; yet they lack the leadership skills to achieve this goal. This perspective discusses the role of student-run free clinics in developing medical students’ leadership abilities—problem-solving, partnership building, planning, decision-making, and resource acquisition—to address the healthcare needs of marginalized patient populations. It also discusses how fostering leadership skills in the context of serving underserved patients also develops medical students’ structural competency and thus awareness of how inequities embedded within hierarchies and social institutions shape health outcomes. We use the example of the development of the Coachella Valley Free Clinic, a student-led and community engaged primary care clinic, to illustrate how student-run free clinics create opportunities for medical students to build leadership skills while addressing the healthcare needs of marginalized patient populations. Medical students, working alongside community health workers and federally qualified healthcare centers, devised a “pop-up” clinic model aimed at delivering care that is both culturally sensitive and linguistically appropriate, thereby addressing health disparities rooted in systemic inequality. As we argue, SRFCs create real-world settings where medical students can develop their leadership skills and understanding of inequities in health ultimately contributing to the broader goal of reducing health inequities by improving healthcare access for underserved patient populations.

## 1 Introduction

Providing healthcare to medically underserved communities is a common thread in medical education. Due to the contextual specificity and dynamic nature of the challenges facing these communities, it can be difficult for students to gain the necessary skill sets to address these problems in a classroom context. Student-run free clinics (SRFCs) present a unique opportunity for medical students to develop essential leadership skills and structural competence while addressing healthcare barriers faced by underserved patient populations in real time (1).

It is well known that the education and socialization process of medical students contributes to declines in empathy and attitudes toward underserved patients (2). This effect is postulated to be the product of a “hidden curriculum” perpetuated by the constellation of clinical and professional experiences that are an inherent, but relatively unregulated, part of modern medical education (3, 4). The opportunities for exposure, agency, and collaboration presented by SRFCs represent a potential challenge to the hidden curriculum of medical education—critical points of intersection that can shift perspectives on the care of underserved patient populations and influence medical school graduates’ intentions to practice in these communities (5, 6). These clinics allow students to engage directly with underserved communities, build partnerships, and develop care models that cater to the needs of vulnerable populations.

SRFCs, although they show great promise, have only recently become regarded as a fundamental component of a comprehensive medical education and are consequently in the early stages of their development as learning and clinical practice spaces (6). Because of their relative youth in the context of medical education, the loose framework that connects these independent organizations is lacking a collective institutional knowledge to buttress the hard work and innovation that lead to their creation (7). In Weinsten et al.'s (2023) study of a network of seven student-run free clinics, the use of educational coaches, a standardized accompanying curriculum, and reflection sessions where clinic volunteers debriefed and processed their clinic experiences were identified as salient improvements that could have universal application in SRFCs. Additionally, peer-to-peer mentorship programs, which result in the creation of site-specific institutional knowledge, have been proposed as a useful improvement to SRFC functioning (8). This commentary uses the development of the Coachella Valley Free Clinic (CVFC) as an example to illustrate how SRFCs help medical students build essential skills while addressing the healthcare needs of the community in the specific context of a critically underserved, rural, Latinx and Indigenous Mexican patient population.

### 1.1 Community health needs and clinic development

The need for the CVFC was identified in a study focused on community health priorities and barriers to healthcare services among Latinx and Indigenous Mexican immigrant farm-working communities in the rural desert region of Inland Southern California (9). This region, home to one of the largest Purépecha–an indigenous group from the Mexican state of Michoacán–communities in the U.S., experiences high levels of poverty, substandard housing, and lack of healthcare access, with many residents being undocumented or uninsured (10). Study findings provided medical students with baseline data on clinic location, service hours, and language and healthcare needs. Medical students partnered with community health workers (CHWs)/promotoras, trusted leaders in the community, and federally qualified healthcare centers (FQHCs) to develop the model of care. The service delivery model was conceptualized as a “pop-up” clinic in a safe community space that provided primary care in the patient's native language, behavioral health screenings and referral, lung health assessments–asthma rates are exceptionally high in the region due to exposure to environmental hazard (11, 12)–and Medicaid sign-ups (13). As detailed in Table 1, the clinic's activities—ranging from mental health screenings to culturally tailored education—yielded significant outcomes, including increased engagement, accessibility, and health awareness among underserved populations. The FQHC contributed to the project by furnishing the clinic with a mobile unit, driver, medical assistant, and clinical faculty preceptor. They also assumed legal responsibility and documented patient care in their electronic medical record system.

**Table 1:** Key indicators of impact: activities, outcomes, and significance of the Coachella Valley Free Clinic.

Indicator	Event/activity	Outcomes	Significance
Community engagement	Suicide prevention presentations (Events in the Eastern Coachella Valley, CA)	Reached 37 community members, with 28 attending presentations. Participants shared personal stories and engaged actively.	Demonstrated interest and trust from vulnerable communities
Mental health screenings	PHQ-9 (depression) and AUDIT-10 (alcohol use disorder) screenings	Conducted screenings during health fairs and distributed resources on mental health and substance use.	Increased awareness of mental health challenges and substance use disorders in underserved populations
Cultural competence	Use of Spanish and Purépecha languages for presentations and discussions	Presentations translated into indigenous languages facilitated engagement.	Enhanced accessibility for monolingual and indigenous community members
Training opportunities	Medical student-led clinics and didactic sessions	25 medical students, 6 physician assistant students, and 25 undergraduates trained in patient-centered care.	Provided skills and mentorship for future healthcare providers in culturally competent care delivery
Healthcare services provided	Coachella Valley Free Clinic (Year 1)	Served 136 patients with medical services, health education, and referrals.	Encouraged engagement in health care within a traditionally underserved population through use of community spaces and collaboration with community health workers
Health education	Workshops for students and community members	Addressed asthma, mental health, and substance use in tailored training sessions	Strengthened the knowledge and intervention skills of both practitioners and community members
Accessibility	Pilot mobile clinics	Clinics tested in different locations and adapted based on community feedback, resulting in increased patient attendance.	Validated flexible, responsive models for healthcare delivery

### 1.2 Medical students’ leadership development

Medical students involved in the CVFC developed key leadership skills through their involvement in the clinic's development and operations. Through their work with CHWs, FQHCs, and bilingual premedical students, they learned to build and maintain relationships and, with faculty mentorship, to access resources through grant funding. Table 1 provides an overview of the activities that fostered leadership skills, such as medical student-led training sessions and tailored educational workshops, which enhanced students’ ability to deliver patient-centered, culturally competent care. Training provided by clinical faculty equipped students with the practical skills needed to deliver care to Spanish- and Purépecha-speaking patients and to disseminate this knowledge to classmates and part-time volunteers. By bringing together this coalition of engaged stakeholders, organizing relevant trainings, and contributing to the structural development of the clinic, students gained valuable expertise and brought a new approach to an intransigent problem. As they navigated this new terrain of decision-making, resource management, and problem-solving, the students developed skills and relationships that colored their perspective on the fluid role of medicine and its practitioners within the broader social context of patient health.

### 1.3 Structural inequities in health

The CVFC highlighted for students how structural inequities shape healthcare access and health outcomes for the patient population, illuminating the ways in which factors such as race/ethnicity, citizenship status, indigeneity, and geography can contribute to the lived experience and health of a specific group of people (10). Structural violence– an inescapable force in immigrant farming communities– often plays out in seemingly ordinary ways, such as clinic hours coinciding with business hours. Positionality within hierarchies of power, which are patterned by social categories (race/ethnicity, gender, citizenship, indigeneity, rurality) determine individual- and population-level vulnerability to structural forces (14). Latinx and indigenous Mexican agricultural workers who reside near the bottom of this hierarchy do not benefit from the protections of regulated work environments and frequently do not have the ability to request time off to attend appointments (15). The experience of interacting with ECV community leaders and members and hearing their first-hand accounts of these barriers faced by the community allowed medical students to recognize the impact of these obstacles on their patients’ ability to seek care. The indicators and outcomes in Table 1 illustrate how the clinic's activities, including health screenings and accessibility improvements, addressed these systemic barriers while providing culturally sensitive care. Through their leadership roles, students addressed these inequities by offering services at times that accommodated agricultural workers’ schedules and providing care in the patients’ primary language.

### 1.4 Innovative models of care

Structural pathologies, by their very nature, necessitate unconventional remedies. If the problem exists in the framework itself, then working within it may not be feasible. Traditional models of healthcare, which rely on brick-and-mortar establishments, often pose barriers for marginalized populations, such as lack of transportation, contingent employment without sick leave, and limited or no insurance coverage (16). The vision and leadership of medical students can result in innovative models of care that challenge these dominant perceptions of “quality” healthcare. The development of the CVFC is a case in point. In partnership with the patient population and FQHCs, medical students developed a model of care involving a walk-up clinic (no appointment needed) in safe and accessible community spaces, with providers who spoke the patient's native language. As shown in Table 1, the clinic's pilot initiatives demonstrated the success of flexible, community-centered care delivery, evidenced by increased attendance and engagement in underserved populations. Providers spent time with each patient, learning about their concerns, how their living and working conditions contributed to their health and well-being, and used this knowledge to tailor treatments—a reversal of the typical model, in which patients unable to adhere to dominant practices are labeled as ‘non-compliant.’

## 2 Conclusion

The CVFC provides a clear example of how SRFCs serve as an effective platform for leadership development for medical students. The clinic's activities and their outcomes, underscore the broader significance of student-run free clinics in addressing health disparities while training future healthcare leaders. By working closely with underserved populations, medical students not only built the leadership skills necessary to manage healthcare delivery in a challenging context, but also developed a deeper understanding of how structural vulnerabilities contribute to health inequities. The innovative model of care developed by the CVFC demonstrates the potential for medical students, with the proper training and partnerships, to create sustainable, patient-centered healthcare solutions that address the unique needs of marginalized and underserved patient populations. These experiences prepare students to become future healthcare leaders who are equipped to navigate the complex social and systemic forces shaping health disparities in patient populations and healthcare access. SRFCs offer medical students the opportunity to lead in real-world settings fostering their leadership skills and contributing to the broader goal of reducing health inequities by improving healthcare access for underserved communities.

## Data Availability

The original contributions presented in the study are included in the article/Supplementary Material, further inquiries can be directed to the corresponding author.
